# Tidal Volume Estimation during Helmet Noninvasive Ventilation: an Experimental Feasibility Study

**DOI:** 10.1038/s41598-019-54020-5

**Published:** 2019-11-21

**Authors:** Andrea Cortegiani, Paolo Navalesi, Giuseppe Accurso, Ignazio Sabella, Giovanni Misseri, Mariachiara Ippolito, Andrea Bruni, Eugenio Garofalo, Cesira Palmeri, Cesare Gregoretti

**Affiliations:** 1 Department of Surgical, Oncological and Oral Science (Di.Chir.On.S.). Section of Anesthesia, Analgesia, Intensive Care and Emergency. Policlinico Paolo Giaccone, University of Palermo, Palermo, Italy; 2 Anesthesia and Intensive Care, Department of Medical and Surgical Sciences, Magna Graecia University, Viale Europa, 88100 Catanzaro Italy

**Keywords:** Therapeutics, Preclinical research

## Abstract

We performed a bench (BS) and human (HS) study to test the hypothesis that estimation of tidal volume (V_T_) during noninvasive helmet pressure support ventilation (nHPSV) would be possible using a turbine driven ventilator (TDV) coupled with an intentional leak single-limb vented circuit. During the BS a mannequin was connected to a lung simulator (LS) and at different conditions of respiratory mechanics, positive end expiratory pressure (PEEP) levels and leaks (30, 50 and 80 L/min). All differences were within the 95% limits of agreement (LoA) in all conditions in the Bland-Altman plot. The overall bias (difference between V_T_ measured by TDV and LS) was 35 ml (95% LoA 10 to 57 ml), 15 ml (95% LoA −40 to 70 ml), 141 ml (95% LoA 109 to 173 ml) in the normal, restrictive and obstructive conditions. The bias at different leaks flow in normal condition was 29 ml (95% LoA 19 to 38 ml). In the HS four healthy volunteers using nHPSV had a pneumotachograph (P) inserted through a mouthpiece to measure subject’s V_T_.The bias showed a scarce clinical relevance. In conclusions, V_T_ estimation seems to be feasible and accurate in all conditions but the obstructive one. Additional leaks seem not to affect V_T_ reliability.

## Introduction

Noninvasive ventilation (NIV) is widely used worldwide to support ventilation and oxygenation in patients with respiratory failure^1^. The helmet is an interface that may increase comfort, reduce skin breakdown, and increase success rate during noninvasive helmet pressure support ventilation (nHPSV) compared to face mask^1–7^.

Measurement of tidal volume (V_T_) still remains an unresolved issue during nHPSV due to the mechanical properties of the helmet^4,6,8–10^. The availability to assess V_T_ during nHPSV might be of importance because it was previously found that high V_T_ during noninvasive ventilation (NIV) in hypoxemic patients may increase lung injury^11–13^. Nevertheless, no study has shown the possibility to assess V_T_ during nHPSV.

Intentional leak single-limb vented circuit configuration allows during face mask or nasal ventilation to make an estimation of the delivered V_T_ in all bi-level ventilators as well as in some intensive care unit (ICU) ventilators mainly dedicated for NIV use^14–16^.

We hypothesized that a turbine driven ventilator (TDV) coupled with intentional leak single-limb vented circuit and whose leak location is set at the helmet expiratory port would allow to estimate patient’s V_T_ during nHPSV^15,16^. The aim of the present study was to test this hypothesis on a bench and healthy volunteer study.

## Methods

The Ethics Committee approved the study protocol (Comitato Etico Palermo 1 – Approval number 07/2018). All experiments were performed in accordance with relevant guidelines and regulations. The study was performed at the Simulation Lab of the Department of Surgical, Oncological and Oral Science (Di.Chir.On.S.), Section of Anesthesia, Analgesia, Intensive Care and Emergency, Policlinico Paolo Giaccone, University of Palermo, Italy.

### Materials and settings

#### Bench study

A modified mannequin head (LaerdalMedical AS, Stavanger, Norway) was connected to a lung simulator (LS) (ASL 5000; Ingmar Medical, Pittsburgh, PA, USA). The ASL 5000 is a digital controlled real-time breathing lung simulator, which allows creating various types of spontaneous breathing pattern and different conditions of respiratory mechanics (e.g. normal, restrictive or obstructive condition). Its functioning is based on a direct-drive screw-driven piston, which moves inside a cylinder according to the equation of motion of an active respiratory mechanics system^17^. Its settings during the bench study, using a single-compartment model, was as follows:

(1) Normal condition: resistance 4 cmH_2_O/L/s, compliance 60 mL/cmH_2_O, inspiratory muscle pressure (Pmus) -5 cmH_2_O (semisinusoidal waveform with rise time of 25%, inspiratory hold of 5%, release time of 25%) and LS respiratory rate set at 15 breaths/min;

(2) Obstructive condition: resistance 15 cmH_2_O/L/s, compliance 80 mL/cmH_2_O, Pmus **-**12 cmH_2_O (semisinusoidal waveform with rise time of 20%, inspiratory hold of 5%, release time of 30%) and LS respiratory rate set at 25 breaths/min;

(2) Restrictive condition: resistance 7.5 mH_2_O/L/s, compliance 30 mL/cmH_2_O, Pmus **-**12 cmH2O (semisinusoidal waveform with rise time of 25%, inspiratory hold of 5%, and release time of 25%), and LS respiratory rate set at 30 breaths/min.

**Figure 1:**
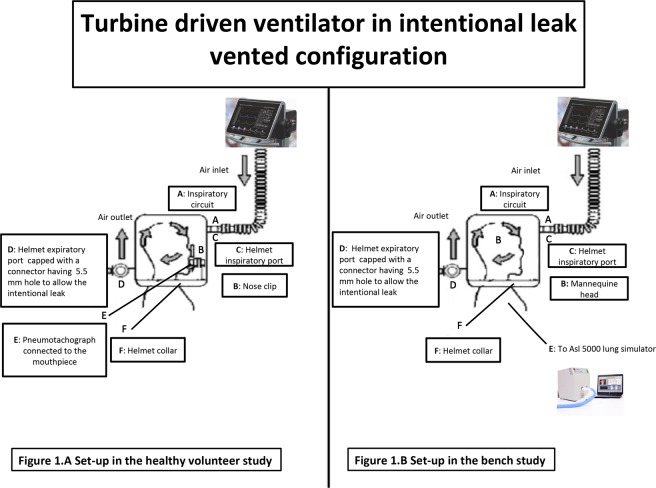
Schematic representation of the experimental set up in the healthy volunteer (**A**) and in the bench study (**B**). Authors’ own figure.

The mannequin head was connected to the LS through the mannequin’s trachea after having positioned and secured a helmet (Castar, Next Intesurgical, size small, Mirandola, Italy)^18^. The helmet inspiratory port was connected to the TDV (Bellavista 100 ICU, Buches, Switzerland) via a single-limb circuit while the expiratory port was closed with a cap having a 5.5 mm hole to allow the intentional leak. This configuration was not provided with any type of inspiratory or expiratory valves and carbon dioxide flushes, as a function of mask pressure through an intentional leak port set in the circuit or in the interface itself. Neither inspiratory nor expiratory V_T_ is measured but they are estimated taking into account the leak according to a mathematical leak model as a function of mask pressure. This mathematical model is used to make an estimation the instantaneous leak and flow which are subtracted from the total flow output of the ventilator (which is the only measured flow) to calculate V_T_^19^. Figure 1 shows the set-up.

During nHPSV, the ventilator was set at the fastest pressure rise time, cycling-off flow threshold of 25% in the normal and restrictive condition and 40% in the obstructive one. Inspiratory flow trigger initially set at 2 L/min was then always tuned at the lowest value not determining auto-triggering^20^. Inspiratory pressure above positive end expiratory pressure (PEEP) was set to reach a V_T_ of about 300/500 ml in all conditions and did not change throughout each condition. Each condition was simulated at PEEP of 5, 8, 10 cmH_2_O. PEEP 12 cmH_2_O was only used in the restrictive condition. During nHPSV leaks were avoided by accurately fit the helmet to the mannequin’s neck. During normal condition at PEEP 8 cmH_2_O and inspiratory pressure (above PEEP) 0 cmH_2_O we also tested the reliability of V_T_ estimation at different leak flows (30, 50 and 80 L/min) using a calibrated hole. During normal condition at PEEP 8 cmH_2_O we also tested the reliability of V_T_ estimation at different leak flows (30, 50 and 80 L/min) using a calibrated hole.

#### Healthy volunteers study

After approval from our Institutional Ethics Committee (Comitato Etico Palermo 1 – approval N° 07/2018) and after obtaining written informed consent, four healthy volunteers, 1 female, 3 male, mean age 33 ± 4 years, mean body weight 70 ± 15 kg were ventilated in nHPSV (PSV 8 cmH_2_O and PEEP 8 cmH_2_O) via a helmet (Castar, Next Intesurgical, size small or medium, Mirandola, Italy), using the same TDV configuration used in the bench study. all experiments were performed in accordance with relevant guidelines and regulations. A mouthpiece was inserted in the volunteer’s mouth and connected to pneumotachograph (P) (V_T_ mobile FLUKE, Germany) to measure subject’s airflow and V_T_. A nose clip was positioned on volunteer’s nostrils to avoid leaks from the nose. The set-up is showed in Fig. 1. All the healthy volunteers were trained, using a metronome, to maintain an imposed respiratory rate of about 12/15 breaths per minute^20^. During nHPSV, ventilator was set at the fastest pressure rise time and at cycling-off flow threshold of 25%. Inspiratory flow trigger was set at the lowest value not determining autotriggering starting from 2 L/min. During nHPSV, leaks were accurately avoided choosing the right helmet size and by appropriately fitting the helmet to the volunteer’s neck.

### Measurements

#### Bench study

Data were collected by the TDV and by LS software. Airflow (V.), the airway pressure (Paw), PEEP as well as the V_T_ and respiratory rate delivered to mannequin were collected over 3 minutes. Differences in V_T_ between TDV and LS were compared during the last 20 breaths of each trial to ensure the stability of the system across settings modification (e.g. increasing, mechanical pattern and pressure level)^16^. During the test at different leak flows, each measurement was performed after 1 minutes after the institution of the new leak to let the TDV to adapt to the new setting. Autotriggering was determined as a mechanical insufflation in absence of inspiratory effort on the LS^17^.

#### Healthy volunteers

Each experimental condition was maintained for 3 minutes, to ensure the stability of the system across the modification of settings (e.g. increasing leak, respiratory pattern)^16^. Paw, PEEP obtained from the TDV and V_T_ and respiratory rate of both TDV software and pneumotacograph (P) were collected. V_T_ measured by TDV and P were compared during the last 20 breaths of each trial.

### Statistical analysis

Normality of data distribution was checked graphically and by D’Agostino-Pearson test. Data are expressed as mean ± standard deviation (SD) or as median and interquartile range (IQR) when appropriate. Bland-Altman graphs were used to plot differences between the V_T_ from the TDV and LS against the average of the two measurements.

Bland-Altman plot is the standard graphical statistical method to evaluate the agreement between quantitative measurements from two different devices, assays or raters. Bland-Altman plots reported the bias (defined as the average of the differences between measurements) and 95% limit of agreements (LoA), namely ± 1.96 SD of the bias^21^. We assumed as clinically relevant a difference of >15% of the LS.

Differences between V_T_ (TDV-LS) at different PEEP levels or leaks were compared using ANOVA for repeated measures and Student-Newman-Keus test was used for all pairwise comparisons. Paired sampled t-test was used to compare V_T_ measured by V and P in the healthy volunteers. P values** < **0.05 were considered statistically significant. We used Prism 7 (GraphPad software; San Diego, CA) and Microsoft Excel (version 2013; Microsoft corporation, Redmond, CA).

## Results

### Bench study

#### Normal, restrictive, obstructive condition

The level of nHPSV to reach the default V_T_ was 8, 10 and 12 cmH_2_O respectively in the normal, restrictive and obstructive condition.

**Figure 2:**
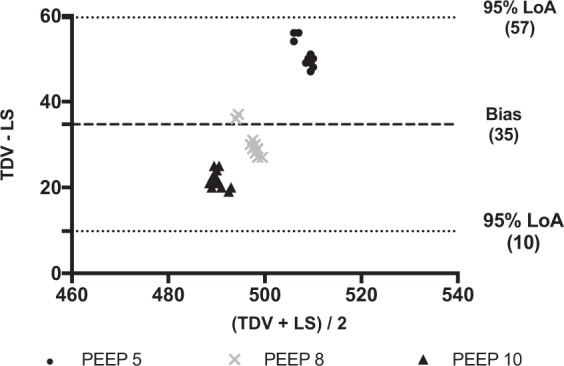
Bland-Altman plot of the differences between tidal volumes measured by the turbine driven ventilator and the lung simulator in the normal condition. Data are reported in ml. PEEP: Positive end expiratory pressure (values are expressed in cmH_2_O). TDV: Tidal volume (V_T_) measured by turbine driven ventilator; LS: Tidal volume (V_T_) measured by lung simulator. Average (x-axys): average of the two measurements; TDV – LS (y-axys): difference between mesurements. Bias: Average of the differences between measurements. 95% LoA: Limits of agreement (±1.96 standard deviation - SD - of the bias). All plotted differences are within the 95% LoA. In this plot, the overall bias is 35 ml, and 95% LoA are from 10 to 57 ml. It reaches the least clinical relevance at PEEP 10 cmH_2_O.

**Figure 3:**
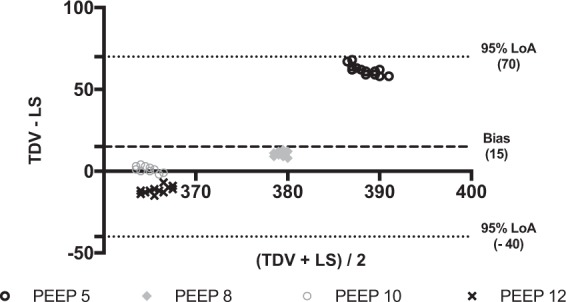
Bland-Altman plot of the differences between tidal volumes measured by the turbine driven ventilator and the lung simulator in the restrictive condition. Data are reported in ml. PEEP: Positive end expiratory pressure (values are expressed in cmH_2_O). TDV: Tidal volume (V_T_) measured by turbine driven ventilator; LS: Tidal volume (V_T_) measured by lung simulator. Average (x-axys): average of the two measurements; TDV – LS (y-axys): difference between mesurements. Bias: Average of the differences between measurements. 95% LoA: Limits of agreement (±1.96 standard deviation - SD - of the bias). In this plot, the overall bias is 15 ml, and 95% LoA are from -40 to 70 ml. All plotted differences are within the 95% LoA. The bias reaches the least clinical relevance at PEEP 10 and 12. It significantly increased at PEEP 5 cmH_2_O.

**Figure 4:**
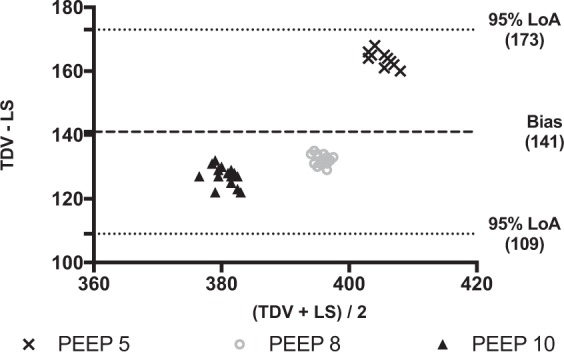
Bland-Altman plot of the differences between tidal volume measured by the turbine driven ventilator and the lung simulator in the obstructive condition. Data are reported in ml. PEEP: Positive end expiratory pressure (values are expressed in cmH_2_O).TDV: tidal volume (V_T_) measured by turbine driven ventilator; LS: Tidal volume (V_T_) measured by lung simulator. Average (x-axys): average of the two measurements; TDV – LS (y-axys): difference between mesurements. Bias: Average of the differences between measurements. 95% LoA: Limits of agreement (±1.96 standard deviation - SD - of the bias). In this plot, the overall bias is 141 ml, and 95% LoA are from 109 to 173 ml. Although all plotted differences are within the 95% LoA, a large clinical relevance bias is found at all tested PEEP levels.

Differences in V_T_ between TDV and LS at different levels of PEEP and pairwise comparisons are shown in Table 1. All plotted differences were within the 95% LoA in all conditions. The overall bias was 35 ml (95% LoA 10 to 57 ml), 15 ml (95% LoA -40 to 70 ml), 141 ml, (95% LoA 109 to 173 ml) respectively in the normal restrictive and obstructive conditions (Figs. 2–4). In the normal condition, the bias reached the least clinical relevance at PEEP 10 cmH_2_O (Fig. 2) while in the restrictive one at PEEP 10 and 12 cmH_2_O (Fig. 3). In the obstructive condition, the bias increased at PEEP 5 cmH_2_O (Fig. 4). Autotriggering was never detected. Tables S1–S3 in the supplementary file 1 reported average V_T_ at different PEEP levels in the different simulated conditions.

**Table 1:** Differences in tidal volumes (V_T_) measured by turbine driven ventilator and lung simulator at different levels of PEEP in the bench study.

Simulated Condition	(TDV-LS) PEEP 5 cmH_2_O	(TDV-LS) PEEP 8 cmH_2_O	(TDV-LS) PEEP 10 cmH_2_O	(TDV-LS) PEEP 12 cmH_2_O	P value
Normal	51 (3)°^+^	31.1(3.5)*^+^	21.8 (2)*°		<0.001
Restrictive	61 (3)°^+§^	10,4 (1.3)*^+§^	1,1 (1.6)*°^§^	−11,9 (1.9)*°^+^	<0.001
Obstructive	164 (2)°^+^	132,5 (1.6)*^+^	127,3(3.1)*°		<0.001

Data are expressed in ml and reported as mean (±SD).

PEEP: positive end expiratory pressure; TDV: turbine driven ventilator; LS: lung simulator; (TDV-LS): difference between V_T_ measurements by turbine driven ventilator and lung simulator.

*Different from 5; °different from 8, ^+^different from 10, ^§^different from 12.

#### Leaks simulation

**Figure 5:**
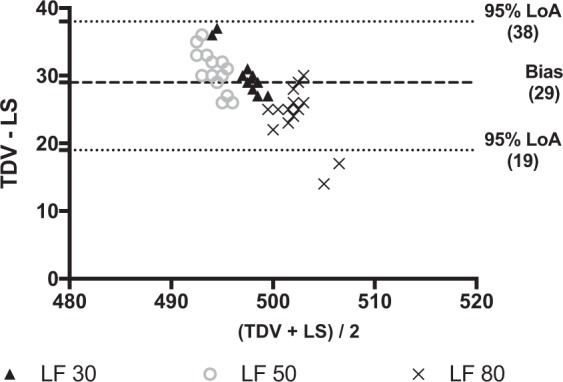
Bland-Altman plot of the differences between tidal volume measured by the turbine driven ventilator and the lung simulator at PEEP 8 cmH_2_O and different levels of leak flow. Data are reported in ml. LF: leak flow (values are expressed in liter per minute); TDV: Tidal volume (V_T_) measured by turbine driven ventilator; LS: Tidal volume (V_T_) measured by lung simulator. Average (x-axys): average of the two measurements; TDV – LS (y-axys): difference between mesurements. Bias: Average of the differences between measurements. 95% LoA: Limits of agreement (±1.96 standard deviation - SD - of the bias). In this plot, the overall bias is 29 ml, and 95% LoA are from 19 to 38 ml. The difference between V_T_ measured by the TDV and the LS remains of no clinical relevance among all simulated leak flows.

Data on the difference in V_T_ between TDV and LS at different levels of leak flow are shown in Table 2. The only significant differences were found between 80 L/min *vs*. 30 L/min and 80 L/min *vs*. 50 L/min. The overall bias was 29 ml (95% LoA 19 to 38 ml) (Fig. 5). Table S4 in the supplementary file 1 reported average V_T_ at different levels of leak flow.

**Table 2:** Differences in tidal volumes (V_T_) measured by turbine driven ventilator and lung simulator at different levels of leak flow.

Simulated Condition	(TDV-LS) LF 30 L/min	(TDV-LS) LF 50 L/min	(TDV-LS) LF 80 L/min	P value
Normal	31(4)^+^	31(3)^+^	24(4)*°	<0.001

Data are expressed in ml and reported as mean (±SD).

LF: leak flow (expressed in liters per minute); TDV: turbine driven ventilator; LS: lung simulator; (TDV-LS): difference between V_T_ measurements by turbine driven ventilator and lung simulator.

*Different from 30; ^°^Different from 50; ^+^Different from 80.

Autotriggering was never detected in any experimental records but at the beginning of the new leak condition before the complete TDV compensation (e.g. switching leak from 30 to 50 L/min).

### Healthy volunteers study

Table 3 shows V_T_ measured by the TDV and the P for the four health volunteers. Mean difference between V_T_ measured by TDV and P was 45 ml (±32) for volunteer 1, 11 ml (±38) for volunteer 2, -35 ml (±37) for volunteer 3 and 58 ml (±40) for volunteer 4. Figure S1 in the supplementary material shows the distribution of V_T_ measured by TDV and P for each volunteer.

**Table 3:** Tidal volumes (V_T_) measured by turbine driven ventilator and pneumotacograph in the human study.

Volunteer N°	TDV V_T_ (ml)	P V_T_ (ml)	P value
1	651 (62.5)	606 (52.8)	<0.001
2	795 (49.1)	784 (38.5)	0.22
3	911 (89.3)	945 (67.1)	0.03
4	697 (74.2)	693 (97.2)	<0.001

All measurements were done at PEEP and nHPSV of 8 cmH_2_O. Data are expressed in ml and reported as mean ± SD; PEEP: positive end expiratory pressure; TDV: V_T_ measured by turbine driven ventilator; P: V_T_ measured by pneumotachograph.

## Discussion

The main findings of the bench study are: (1) No clinically relevant differences were found in V_T_ between TDV and LS in normal and restrictive conditions at all simulated level of PEEP; (2) The difference between TDV and LS remained stable across the different tested leak flows; (3) There was a large, clinically relevant, difference in V_T_ in the obstructive condition between TDV and the LS.

To the best of our knowledge this is the first study able to demonstrate the possibility of estimate V_T_ during helmet ventilation. The same circuit configuration was tested in a previous study, which used nHPSV in single-circuit vented intentional leak configuration to measure carbon dioxide (CO_2_) rebreathing^15^. Interestingly, although V_T_ was not measured by the ventilator software in this study, this kind of circuit configuration should theoretically allow to estimate V_T_ as during non invasive intentional leak mask ventilation^16^. This estimation has been demonstrated to be pretty reliable in different condition of respiratory mechanics and linear non intentional leaks^16^.

Our bench study was carried out with a TDV with an intentional leak vented single-limb circuit because direct measurement of V_T_ was not possible due to the intrinsic mechanical features of the helmet, namely its large internal volume^15^. Besides, a TDV with an intentional leak vented single-limb circuit better compensate for air leaks up to its leak compensation capability^16,22^.

The helmet is an interface that may increase comfort, reduce skin breakdown, and increase success rate during nHPSV compared to face mask^1–7^. The TDV was coupled to a new commercially available helmet. This new helmet is featured by an annular extendable ring placed under an inflatable large cushion that secures the interface without the need for armpit support^23^. This helmet has been found to reduce to a large extent its upward displacement during the ventilator support thus improving patient–ventilator interaction^23^.

Our results found a very good agreement between differences in V_T_ estimated by the TDV and that measured by the LS in the normal and restrictive condition using the Bland Altman plot. Although the difference between V_T_ measured by the LS and the TDV was statistically different among the groups at the different PEEP levels, this difference was of poor clinical relevance. Of note, Lyazidi *et al*. found that when setting a V_T_ of 6 ml/kg of predicted body weight, a difference of 1–2 ml/kg with the actually delivered V_T_ would be commonly found^24^.

We found, both in the normal and in restrictive condition, that the higher the PEEP the less the difference between the V_T_ measured by the TDV and the LS. This result is very appealing being the restrictive lung one the most common application of helmet ventilation^4,25^. Conversely, in the obstructive condition we found, although still in the 95% LoA, a clinically relevant discrepancy between the two V_T_ with an overestimation showed by the TDV. Interestingly, as opposite to our findings, Lujan *et al*. found that built-in software was underestimating the V_T_ in two of the tested ventilators^16^. This may suggest that different built-in software may behave differently in presence of an obstructive pattern. One can also speculate that understimation might be linked to the reassessment of the zero flow value by the TDV in presence of air trapping. However, we can also hypothesize that during the bench study in this condition, the simulated patient’s high resistances could have generated small non intentional leaks. While the ventilator software was not affected by the presence of the leak because it was able to compensate it, the monitoring system of the ASL 5000 did so reading smaller V_T_.

Autotriggering was never detected in any trial. This result supports previous finding of the TDV capability in intentional leak vented configuration to better compensate for leaks when a flow trigger is used^22^. Autotriggering could also not be found due to the use of the new helmet, which presents less upward displacement during nHPSV. Only when simulated a new leak flow, few auto-triggered breaths were detected due to the need of the ventilator to compensate the loss in air flow.

Non intentional leaks are the most frequent drawback during NIV^26^. We evaluated the reliability of V_T_ estimation increasing the values of the intentional leak flows to 30, 50 and 80 L/min during nHPSV in the normal simulated condition at PEEP of 8 cmH_2_O. This range was selected because it includes the minimal value to avoid rebreathing even in absence of the inspiratory support^7^. Again, the difference in V_T_ at different leak flows was clinically negligible.

Software may underestimate both leaks and V_T_ with the greater the leak, the greater the difference between the estimated and actual V_T_^27^. However, the ventilator software we used was provided with an algorithm that improves accuracy compensating for the difference between the estimated leak measured at the distal and proximal sides of the circuit^16^. The interesting clinical message is that being leaks a possible drawback of helmet ventilation this way of estimating V_T_ could theoretically assures V_T_ estimation even in presence of additional leaks.

The main findings of the healthy volunteer study are that although the differences in V_T_ were significantly different in all the volunteers but the second one, this difference was of scarce clinical relevance. During the human study, we used the same set-up that we previously described in other study^15^. We applied PEEP at 8 cmH2O because it is one of the most common value used in the clinical setting^18,28,29^.

Estimation of V_T_ has been the only mode of measuring V_T_ in TDV in intentional leaks single-limb vented configuration since the introduction of these ventilators at the beginning of the 90^16,27^. The built-in software of these TDV should be able to accurately estimate V_T_, even in presence of increased non-intentional leaks. Of note, using pressure controlled modes with compressed air driven intensive care unit (ICU) ventilator, the higher the non intentional leak the lower the expiratory V_T_^22,30^. In addition, by using an helmet, which has a larger internal volume, the displayed V_T_, when using a non vented configuration with a high pressure driven ICU ventilator is significantly higher than actual delivered V_T_^5^.

We can speculate that, using a TDV in this configuration, it would be possible to ventilate hypoxemic patients^4^ via an helmet by knowing that delivered V_T_^11^ is pretty closed to what the patients is really breathing in.

The present study has several limitations. Firstly, this is a bench and human volunteers study. Therefore, these results need to be confirmed clinically. However, direct comparison of real measurements and its estimation at standardized condition of respiratory mechanics and respiratory rate in the same patient seems impossible for both practical and ethical reasons. Moreover, it should be noted that volunteers had healthy lungs. Second, we measured V_T_ under ambient temperature and pressure and dry gas conditions (ATPD). However, the V_T_ in the patient’s lungs is at body temperature and pressure, and saturated with water vapor (BTPS). The conversion from ATPD to BTPS would increase V_T_ by about 10.4% for TDVs at 20 °C and at the vapor pressure of H_2_O 17.5 mmHg^16^. We could speculate that the small gap we found between TDV and LS would be potentially further reduced by converting ATPD in BTPS. Third, we used to simulate an increase in leaks an increase in linear non intentional leak^31^. As previously demonstrated by Sogo *et al*., the presence of unintentional random leaks may lead to an error in the measurement of V_T_ provided by the ventilator software^31^. In addition, we did not measure the pressurization time at the different simulated leaks. It is possible that by increasing the amount of the leak (intentional or non intentional) we approached the compensating capability of the TDV thus increasing the time necessary to achieve the preset level of pressure support^5^. Fourth, we did not measure CO_2_ rebreathing in the human study. However, we previously demonstrated that the same setting of the present study improved CO2 rebreathing compared to a non-vented circuit configuration using an air compressed ICU ventilator^15^. Lastly, due to different algorithms used to estimate V_T_ in the build-in software of TDV, we cannot be sure that our data can be extrapolated to other TDVs in vented configuration^16,27^.

In conclusion, this feasibility bench and human study demonstrated that V_T_ estimation during nHPSV seems to be feasible and accurate in all the conditions except the simulated obstructive condition. Additional leaks seem not to affect V_T_ reliability. Further studies are required to identify the magnitude of discrepancy between the proposed method and gold standard in bench and clinical setting.

## Supplementary information

Additional file 1

## Data Availability

The datasets analysed during the current study are available from the corresponding author on reasonable request.
