# Supplementary material for: Tidal Volume Estimation during Helmet Noninvasive Ventilation: an Experimental Feasibility Study
Source: Sci Rep. 2019 Nov 21;9:17324. doi: 10.1038/s41598-019-54020-5 (PMC6872634; doi:10.1038/s41598-019-54020-5)
Supplement: Supplementary file 1 — Additional file 1 [file 41598_2019_54020_MOESM1_ESM.pdf]

Supplementary material

Tidal volume estimation during helmet noninvasive ventilation: an experimental feasibility study

Andrea Cortegiani[1], Paolo Navalesi[2], Giuseppe Accurso[1], Ignazio Sabella[1], Giovanni Misseri[1], Mariachiara Ippolito[1], Andrea Bruni[2], Eugenio Garofalo[2], Cesira Palmeri[1] and Cesare Gregoretti[1]

[1]Department of Surgical, Oncological and Oral Science (Di.Chir.On.S.). Section of Anesthesia, Analgesia, Intensive Care and Emergency. Policlinico Paolo Giaccone. University of Palermo, Italy.

[2]Anesthesia and Intensive Care, Department of Medical and Surgical Sciences, Magna Graecia University, Catanzaro, Italy.

Table S1. Avarage tidal volumes (V_T) measured by the turbine driven ventilator and the lung simulator at different levels of PEEP in simulated normal condition.

	**TDV**	**LS**
**PEEP 5**	534 ± 1	483 ± 3
**PEEP 8**	513 ± 1	481 ± 3
**PEEP 10**	501 ± 1	480 ± 1

Data are expressed in ml and reported as mean ± SD.
PEEP: positive end expiratory pressure (in cmH_2O); TDV: turbine driven ventilator; LS: lung simulator.

Table S2. Avarage tidal volumes (V_T) measured by the turbine driven ventilator and the lung simulator at different levels of PEEP in simulated restrictive condition.

	**TDV**	**LS**
**PEEP 5**	419 ± 1	358 ± 2
**PEEP 8**	384 ± 1	374 ± 1
**PEEP 10**	365 ± 1	364 ± 1
**PEEP 12**	360 ±2	371 ± 2

Data are expressed in ml and reported as mean ± SD.
PEEP: positive end expiratory pressure (in cmH_2O); TDV: turbine driven ventilator; LS: lung simulator.

Table S3. Average tidal volumes (V_T) measured by the turbine driven ventilator and the lung simulator at different levels of PEEP in simulated obstructive condition.

	**TDV**	**LS**
**PEEP 5**	487± 1	323 ± 3
**PEEP 8**	462 ± 1	329 ± 2
**PEEP 10**	444± 1	317 ± 3

Data are expressed in ml and reported as mean ± SD.
PEEP: positive end expiratory pressure (in cmH_2O); TDV: turbine driven ventilator; LS: lung simulator.

Table S4. Average tidal volumes (V_T) measured by the turbine driven ventilator and the lung simulator at different leak flows.

	TDV	LS
**LF 30**	513 ± 1	481 ± 3
**LF 50**	510 ± 1	479 ± 2
**LF 80**	515 ± 2	490 ± 3

Data are expressed in ml and reported as mean ± SD.
LF: leak flow (expressed in liters per minute); TDV: turbine driven ventilator; LS: lung simulator.

Figure S1. Distribution of tidal volumes (V_T) measured by the turbine driven ventilator and the pneumotachograph for each healthy volunteer.

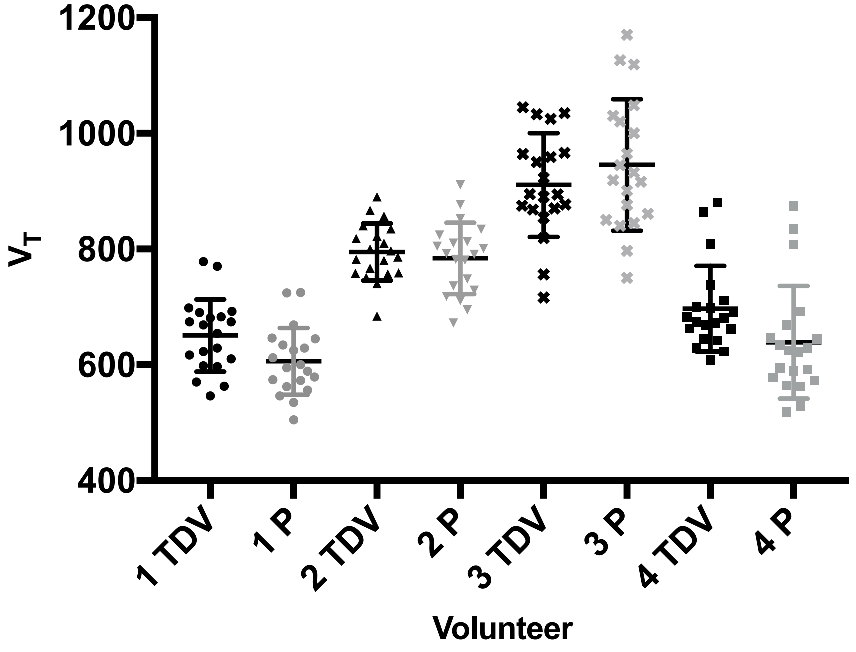

V_T**:** Tidal volume (in ml). Measurement by the turbine driven ventilator (TDV) and by the pneumotachograph (P) for each volunteer are reported with the same legend but different color (black - TDV, grey – LS). Bars describe mean ± SD.
